# Beyond Cannabidiol: The Contribution of *Cannabis sativa* Phytocomplex to Skin Anti-Inflammatory Activity in Human Skin Keratinocytes

**DOI:** 10.3390/ph18050647

**Published:** 2025-04-28

**Authors:** Marco Fumagalli, Giulia Martinelli, Giuseppe Paladino, Nora Rossini, Umberto Ciriello, Vincenzo Nicolaci, Nicole Maranta, Carola Pozzoli, Safwa Moheb El Haddad, Elisa Sonzogni, Mario Dell’Agli, Stefano Piazza, Enrico Sangiovanni

**Affiliations:** 1 Department of Pharmacological and Biomolecular Sciences “Rodolfo Paoletti”, Università degli Studi di Milano, 20133 Milan, Italy; marco.fumagalli3@unimi.it (M.F.); giulia.martinelli@unimi.it (G.M.); vincenzo.nicolaci@unimi.it (V.N.); nicole.maranta@unimi.it (N.M.); carola.pozzoli@unimi.it (C.P.); safwa.elhaddad@guest.unimi.it (S.M.E.H.); elisa.sonzogni@unimi.it (E.S.); mario.dellagli@unimi.it (M.D.); enrico.sangiovanni@unimi.it (E.S.); 2 Linnea SA, 6595 Riazzino, Switzerland; gpaladino@linnea.ch (G.P.); nrossini@linnea.ch (N.R.); uciriello@linnea.ch (U.C.)

**Keywords:** *Cannabis sativa*, skin inflammation, CBD, THC, fractionation, phytocomplex

## Abstract

**Background:** *Cannabis sativa* L. (*C. sativa*) has a long history of medicinal use. Its inflorescences contain bioactive compounds like non-psychotropic cannabidiol (CBD), which is well known for its anti-inflammatory potential in skin conditions such as psoriasis, and psychotropic Δ-9-tetrahydrocannabinol (THC). Keratinocytes, the main cells in the epidermis, are crucial for regulating skin inflammation by producing mediators like IL-8 when stimulated by agents like TNFα. **Methods:** This study explores the anti-inflammatory effects of a standardized *C. sativa* extract (CSE) with 5% CBD and less than 0.2% THC in human keratinocytes challenged by TNFα. The aim of this study is to analyze the specific contributions of the main constituents of CSE to inflammatory responses in human keratinocytes by fractionating the extract and examining the effects of its individual components. **Results:** MTT assays showed that CSE was non-toxic to HaCaT cells up to 50 μg/mL. CSE inhibited NF-κB activity and reduced IL-8 secretion in a concentration-dependent manner, with mean IC_50_ values of 28.94 ± 10.40 μg/mL and 20.06 ± 2.78 μg/mL (mean ± SEM), respectively. Fractionation of CSE into four subfractions revealed that the more lipophilic fractions (A and B) were the most effective in inhibiting NF-κB, indicating that cannabinoids and cannflavins are key contributors. Pure CBD is one of the most active cannabinoids in reducing NF-κB-driven transcription (together with THC and cannabigerol), and due to its abundance in CSE, it is primarily responsible for the anti-inflammatory activity. **Conclusions**: This study highlights CBD’s significant role in reducing inflammation in human keratinocytes and underscores the need to consider the synergistic interactions of several molecules within *C. sativa* extracts for maximum efficacy. Standardized extracts are essential for reproducible results due to the variability in responses.

## 1. Introduction

*Cannabis sativa* L. (*C. sativa*), also known as Indian hemp, is an herbaceous annual plant that has been cultivated in Central Asia, particularly in India and China, for diverse applications. Over the centuries, various parts of the plant have been utilized for medicinal, recreational, and religious purposes, such as extracting healing oils from seeds and using inflorescences for their psychoactive effects [1]. *C. sativa* inflorescences contain a plethora of bioactive compounds, notably cannabinoids such as cannabidiol (CBD) and Δ-9-tetrahydrocannabinol (THC). These cannabinoids have garnered considerable attention for their potential therapeutic benefits in various skin inflammatory conditions [2,3]. CBD, the second major cannabinoid found in *C. sativa*, is particularly valued for its lack of psychotropic effects, unlike its counterpart, THC. Cannabinoids interact with specific receptors in keratinocytes, such as CB1, CB2, TRPV1, TRPV3, and TRPA1, influencing various cellular functions and responses [4]. However, studies have indicated that other constituents of cannabis, such as terpenes [5], cannflavins [6,7], and canniprene [8],, may also contribute to its anti-inflammatory effects, although their roles are less well understood than those of CBD and THC. In dermatological inflammation, keratinocytes, the predominant cell type in the epidermis, play a pivotal role in initiating and perpetuating inflammatory responses. These cells can release a wide range of inflammatory mediators, including IL-8 [9] when stimulated with cytokines typically associated with dermatitis, such as tumor necrosis factor-alpha (TNFα) [10]. IL-8 belongs to the CXC chemokine family and plays a crucial role in mobilizing leukocytes toward areas of inflammation. Typically, cells in a non-stimulated state produce negligible levels of IL-8; however, its expression can be swiftly triggered by various inducers, including pro-inflammatory cytokines, microbial agents, and stress from cellular mechanisms [11]. The regulation of IL-8 is strictly related to the activation of the nuclear transcription factor κB (NF-κB) pathway and occurs at both the transcriptional and post-transcriptional stages.

The role of *Cannabis* in skin health is currently under investigation in the context of psoriasis, UV damage, wound healing, melanoma, and acne [12,13,14]. Our previous research demonstrated the anti-inflammatory properties of a standardized ethanolic extract from *C. sativa*, containing 5% CBD and a low concentration of THC (≤0.2%), in human keratinocytes [15]. This extract was found to modulate inflammation by downregulating pro-inflammatory genes and inhibiting NF-κB activity. CBD has been reported as one of the most promising non-psychotropic cannabinoids for skin inflammation, and its mechanism of action involves NF-κB impairment [4,16]; however, its interaction with other phytochemicals present in *C. sativa* extracts is still a matter of debate.

Given the complexity of cannabis constituents and their potential interactions, the specific contributions of individual cannabinoids and other compounds, like terpenes and cannflavins, to anti-inflammatory activity require further elucidation.

Therefore, the aim of the present study was to investigate the specific role of the main constituents of a *C. sativa* extract in the biological activities of human keratinocytes, focusing on their potential to modulate inflammatory responses. Indeed, by fractionating the extract and analyzing the effects of its individual components, this study endeavors to provide a clearer understanding of the interaction between CBD and minor compounds belonging to the *C. sativa* phytocomplex. This research builds on foundational work on CBD that established the basic anti-inflammatory effects of cannabis extracts in keratinocytes, driving further investigations into their therapeutic potential.

## 2. Results

### 2.1. Cytotoxicity and Anti-Inflammatory Effects of CSE

**Figure 1:**
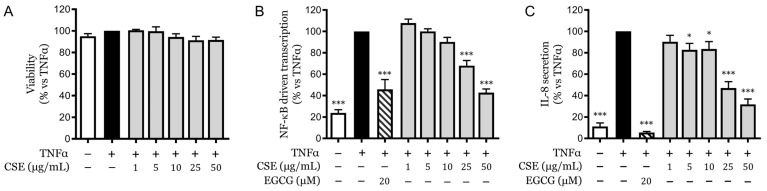
Cytotoxicity and anti-inflammatory effects of CSE on HaCaT cells. (**A**) Cell viability was measured by MTT assay after treatment with various concentrations of CSE (0–50 µg/mL). (**B**) NF-κB-driven transcriptional activity in HaCaT cells treated with CSE in the presence of TNFα (10 ng/mL), as measured by luciferase reporter assay. (**C**) IL-8 secretion levels in HaCaT cells treated with CSE in the presence of TNFα, quantified by ELISA. Data are expressed as the mean ± SEM of three independent experiments. * *p* < 0.05, *** *p* < 0.001 vs. TNFα-stimulated control.

Skin inflammatory disorders often involve the overproduction of various pro-inflammatory mediators that significantly affect the function and health of the keratinocyte population. To evaluate the potential effects of CSE, its cytotoxicity and anti-inflammatory efficacy were assessed in HaCaT cells, a commonly used human keratinocyte model. The results obtained by MTT assay, a well-known indicator of cell viability, confirmed that CSE did not exhibit cytotoxic effects at the highest concentration tested, which was 50 μg/mL. This outcome demonstrated that cellular integrity and metabolic function were effectively preserved within this concentration range (Figure 1A).

NF-κB is a pivotal transcription factor implicated in numerous skin inflammatory conditions, including psoriasis [17], and is known to be highly activated by TNFα. Given the critical role of NF-κB in mediating inflammatory responses, the first step of our study focused on examining whether CSE could mitigate NF-κB activation induced by TNFα.

Since the natural compound, EGCG, is a widely recognized anti-inflammatory compound in the skin context [18], it was used as a reference inhibitor in comparison with CSE. In our experiments, CSE displayed concentration-dependent inhibition of NF-κB-driven transcription in HaCaT cells. The half-maximal inhibitory concentration (IC_50_) for this effect was determined to be 28.94 ± 10.40 μg/mL (Figure 1B), which is consistent with the inhibitory concentrations observed in previous samples of CSE [15].

Furthermore, IL-8, a cytokine crucial for the recruitment of neutrophils and a key downstream effector of NF-κB, was also measured to assess the impact of CSE on this pathway. TNFα stimulation markedly increased IL-8 secretion in keratinocytes, which was significantly impaired by CSE in a concentration-dependent manner. The IC_50_ for the reduction of IL-8 secretion by CSE was found to be 20.06 ± 2.78 μg/mL (Figure 1C).

### 2.2. Fractionation and Differential Cytotoxicity

**Figure 2:**
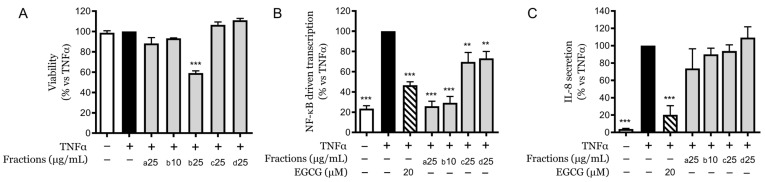
Effects of CSE fractions on viability, NF-κB-driven transcription, and IL-8 secretion in HaCaT cells. (**A**) Cytotoxicity of CSE fractions at 25 µg/mL (or 10 µg/mL) assessed by MTT assay. (**B**) NF-κB-driven transcriptional activity in HaCaT cells treated with non-toxic concentrations of CSE fractions (a–d) in the presence of TNFα, measured by luciferase reporter assay. (**C**) IL-8 secretion levels in HaCaT cells treated with CSE fractions in the presence of TNFα, quantified by ELISA. Data are expressed as mean ± SEM of three independent experiments. ** *p* < 0.01, *** *p* < 0.001 vs. TNFα-stimulated control.

Following the fractionation of the CSE based on increasing polarity, the cytotoxicity and anti-inflammatory effects of the four distinct fractions were examined. Among the fractions studied, Fraction (a) was the most lipophilic, while Fraction (d) was the most polar. Initial cytotoxicity assessment at 25 µg/mL revealed that only Fraction (b) demonstrated cytotoxic effects at this concentration, while it did not exert cytotoxicity at 10 µg/mL (Figure 2A).

### 2.3. Evaluation of Anti-Inflammatory Effects of Fractions at Non-Toxic Concentrations

The fractions were subsequently tested at their maximum non-toxic concentrations for their ability to influence key inflammatory markers, specifically NF-κB-driven transcription and IL-8 secretion. The results indicated that the more apolar Fractions (a) and (b) retained the greatest capacity to inhibit NF-κB transcription. Specifically, Fraction (a) at 25 µg/mL and Fraction (b) at 10 µg/mL reduced luciferase transcription to levels comparable to those of the unstimulated control. Conversely, the more polar Fractions (c) and (d) demonstrated a preserved but less potent inhibitory effect, reducing transcription by approximately 30% relative to the control (Figure 2B).

In terms of IL-8 secretion, none of the fractions significantly inhibit this parameter (Figure 2C), suggesting that the observed inhibitory effects may require the synergistic action of multiple molecules present within the phytocomplex of CSE. Thus, for a better comprehension of this observation, each fraction was chemically characterized.

### 2.4. Analytical Profiling of Fraction Components

**Figure 3:**
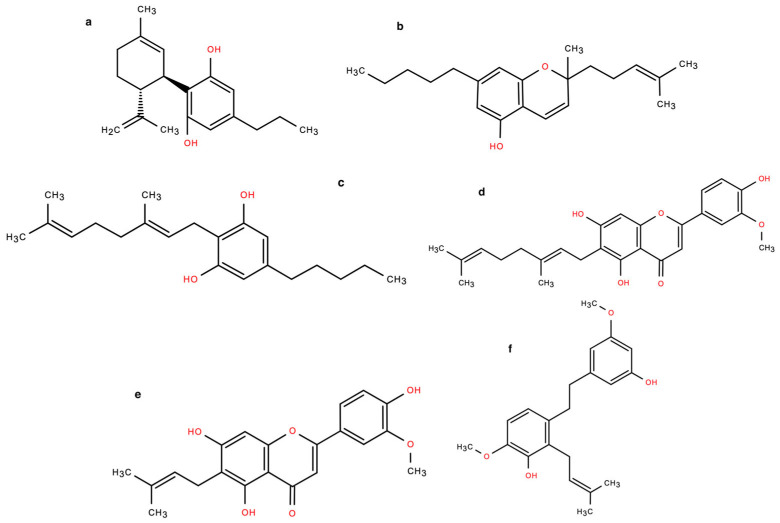
Chemical structure of CBDV (**a**), CBC (**b**), CBG (**c**), CFN-A (**d**), CNF-B (**e**), and CNP (**f**).

An analytical investigation of the composition of these fractions revealed distinct profiles of cannabinoids, terpenes, and cannflavins (Table 1). Fractions (c) and (d) were devoid of cannabinoids and cannflavins and contained only a small residual percentage of terpenes. In contrast, Fractions (a) and (b) were rich in cannabinoids. Notably, Fraction (a) contained more than double the concentration of CBD and nearly nine times the terpene content compared to Fraction (b). It also contained 1% THC, which was absent in Fraction (b). The concentrations of other analyzed cannabinoids, including cannabidivarin (CBDV), cannabichromene (CBC), and cannabigerol (CBG), were found to be comparable across these fractions. Fraction (b), however, was characterized by a higher content of cannflavins, including both Cannflavin A (CFN-A) and B (CFN-B). For better comprehension, the chemical structures of the compounds mentioned above are shown in Figure 3. These results highlight the complexity of the anti-inflammatory effects observed, suggesting a significant role for cannabinoids and terpenes in modulating these responses.

### 2.5. Analysis of Pure Compounds

To clearly identify the compounds responsible for the inhibitory activity on NF-κB transcription, this parameter was chosen to compare the effects of individual molecules. CSE was analyzed as a whole and in terms of its most abundant molecular components, as detailed in Table 2. Among the cannabinoids, as expected, CBD was the most abundant, constituting 4.84% of the extract, followed by CBC at 0.24%, THC at 0.22%, and CBG at 0.14%. Additionally, a detailed analysis was conducted on the terpene blend within the extract, which includes γ-terpineol, β-caryophyllene, α-humulene, and three isomers of farnesene, along with caryophyllene oxide and α-bisabolol. Among them, β-caryophyllene emerged as the most prevalent terpene (0.05% of the extract) (Table 2).

**Figure 4:**
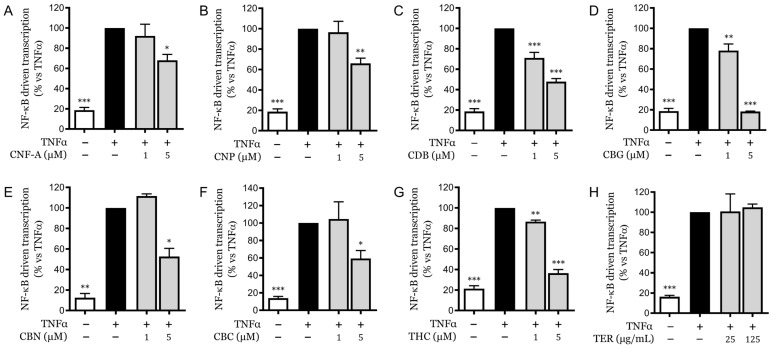
Inhibition of NF-κB-driven transcription by pure cannabinoids and terpenes in HaCaT cells. (**A**–**G**) NF-κB-driven transcriptional activity in HaCaT cells treated with CFN-A, CNP, and various cannabinoids at 5 µM in the presence of TNFα, measured by luciferase reporter assay. (**H**) NF-κB-driven transcriptional activity in HaCaT cells treated with a reconstituted terpene mix at high concentrations in the presence of TNFα. Data are expressed as mean ± SEM of three independent experiments. * *p* < 0.05, ** *p* < 0.01, *** *p* < 0.001 vs. TNFα-stimulated control.

In our assays, CFN-A (Figure 4A), canniprene (CNP) (Figure 4B), and the various evaluated cannabinoids (Figure 4C–G) exhibited inhibitory effects on NF-κB-driven transcription, particularly at 5 µM concentration. None of the molecules or the terpene mixtures showed cytotoxic activity at the tested concentrations (Figure S1). Among these, CBG was the most potent, reducing transcription to levels comparable to that of the unstimulated control (Figure 4D). The mixture of various terpenes (Figure 4H) was reconstituted by adding individual components back into a medium-chain triglyceride (MCT) mixture while maintaining the proportions found in CSE. This blend was tested at concentrations exceeding 500 and 3000 times those present in the extract. Despite the high concentrations, the terpene mixture did not affect NF-κB transcription, suggesting that these molecules do not play a direct role in the observed anti-inflammatory activity. For comparison, 25 µg/mL of CSE corresponds to molarities of 0.051 µM for γ-terpineol, 0.061 µM for β-caryophyllene, 0.015 µM for α-humulene, 0.012 µM for farnesene isomer 1, 0.086 µM for farnesene isomer 2, 0.018 µM for farnesene isomer 3, 0.017 µM for caryophyllene oxide, and 0.012 µM for α-bisabolol. Among the other molecules, only CBD reached significant concentrations within the extract, which can partly account for the inhibition observed with CSE. However, it is important to note that the inhibition achieved by purified CBD on this parameter was greater than that observed with the same concentration of CSE, which contained 3.85 µM of CBD at 25 µg/mL. This suggests the presence of antagonistic interactions within the extract that may modulate its overall bioactivity.

### 2.6. Synergistic Effects of Cannabinoids and Terpenes

Finally, a comparative analysis was carried out between CSE and a reconstituted mixture of its main identified components, predominantly cannabinoids, which reflected their concentrations within the extract. This mixture was prepared in MCT solution, which was also evaluated separately as a control and exhibited no inhibitory effects. The composition of the reconstituted mixture (MIX) included CBD at 3.85 µM, CBG at 0.11 µM, CNP at 0.013 µM, CBC at 0.19 µM, CNF-A at 0.13 µM, cannabinol (CNB) at 0.0008 µM, and THC at 0.16 µM.

**Figure 5:**
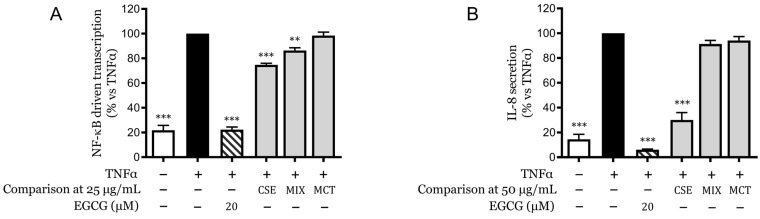
Comparative analysis of CSE and reconstituted mixtures of identified components on NF-κB-driven transcription and IL-8 secretion. (**A**) NF-κB-driven transcriptional activity in HaCaT cells treated with CSE and MIX in the presence of TNFα, measured by luciferase reporter assay. (**B**) IL-8 secretion levels in HaCaT cells treated with CSE and MIX at 50 µg/mL in the presence of TNFα, quantified by ELISA. Data are expressed as mean ± SEM of three independent experiments. ** *p* < 0.01, *** *p* < 0.001 vs. TNFα-stimulated control.

As observed in Figure 5A, the MIX, equivalent to a concentration of 25 µg/mL of CSE, demonstrated a lower reduction in NF-κB-driven transcription than the whole CSE. The same comparison was extended to the secretion of IL-8 (Figure 5B) but at a concentration of 50 µg/mL instead. Here, the MIX displayed a markedly inferior effect compared to CSE, such that it was not statistically significant against the TNFα-stimulated control.

These findings suggest that while individual components of CSE have specific inhibitory effects, their collective interaction within an artificially reproduced mixture can alter the overall bioactivity, potentially due to competitive or antagonistic interactions between the components.

## 3. Discussion

Keratinocytes, the predominant cell type in the epidermis, play a pivotal role in the initiation and regulation of cutaneous inflammatory responses. These cells respond dynamically to various stimuli, including cytokines, microbial agents, and environmental stressors, leading to the production of a broad range of pro-inflammatory mediators, such as IL-8. Cannabinoids [19,20], including CBD and THC [15,21], have been shown to modulate these pathways in keratinocytes, presenting potential therapeutic benefits for managing inflammatory skin conditions.

Cannabinoids, particularly CBD, have demonstrated considerable potential in treating inflammatory skin disorders due to their ability to modulate key inflammatory pathways [3,12,22]. The entourage effect, a synergistic phenomenon in which multiple components of cannabis interact to enhance therapeutic outcomes, is also gaining attention [23]. This suggests that the combined action of cannabinoids and other phytochemicals in cannabis could provide enhanced therapeutic benefits compared to isolated cannabinoids. For example, the combination of CBD and THC has shown promise in reducing neuroinflammation by leveraging their synergistic effects to modulate distinct signaling pathways, thereby offering a more comprehensive therapeutic approach for neuroinflammatory conditions [24].

The topical application of cannabinoids takes advantage of their anti-inflammatory, antioxidant, and analgesic properties, providing localized relief without systemic side effects. Studies have shown that cannabinoids can inhibit the production of pro-inflammatory cytokines and chemokines, modulate keratinocyte proliferation and differentiation, and enhance skin barrier function [25]. Topical cannabinoid application offers several advantages, including targeted delivery to affected areas, reduced systemic absorption, and a minimized risk of the psychoactive effects associated with systemic THC use. However, challenges such as skin permeability, the stability of cannabinoid formulations, and the need for optimal delivery systems must be addressed to maximize efficacy. Innovative delivery systems, like liposomes, nanoparticles, and transdermal patches, are being explored to overcome these limitations [26].

The present study aimed to dissect the specific contributions of the main constituents of CSE to its anti-inflammatory activity in human keratinocytes. The ability of CSE to inhibit both NF-κB activity and IL-8 secretion suggests a beneficial mechanism of action in managing conditions characterized by excessive inflammatory responses, particularly in the skin. This transcription factor plays a central role in several inflammatory skin disorders, including psoriasis [17]. By analyzing the whole extract and its individual fractions, we sought to elucidate the mechanistic pathways through which CSE exerts its effects, focusing on NF-κB-driven transcription and IL-8 secretion. The fractionation of CSE into four subfractions based on increasing polarity indicated that the lipophilic compounds within these fractions, such as cannabinoids and cannflavins, are major contributors to the anti-inflammatory activity of CSE. Detailed analytical profiling revealed that Fractions (a) and (b) were rich in cannabinoids, particularly CBD, which was the most abundant compound. The presence of other cannabinoids, such as CBC and CBG, albeit at lower concentrations, suggests a synergistic effect contributing to overall bioactivity. Our findings confirm the significant role of CBD, among other cannabinoids, in inhibiting NF-κB activation [15], but CBG showed the highest overall inhibition. Surprisingly, our results on pure CBD indicate that other cannabinoids, despite their individual inhibitory properties, may interfere with the activity of CBD in the mixture, possibly through receptor competition or antagonistic interactions. Petrosino et al. suggested that CB2 agonists might reverse the anti-inflammatory activity of CBD at the keratinocyte level [16].

Evaluation of IL-8 secretion revealed that other molecules might contribute to the biological activity observed with CSE. In this case, we excluded the possible contribution of terpenes to the bioactivity, as they were inactive even at high concentrations, and CBD has previously been shown to possess significant activity [15]. The lack of significant IL-8 inhibition by the fractions suggests that the anti-inflammatory effects of the full extract may require the combined action of multiple molecules, reinforcing the complexity of phytochemical interactions. Notably, the reconstituted mixture of CSE’s main identified components of CSE (MIX) did not significantly inhibit IL-8 secretion as effectively as CSE; thus, we cannot exclude the possibility that compounds outside our analytical identification and MIX reconstitution may have contributed to IL-8 inhibition as entourage elements in CBD bioactivity. In addition, several mechanisms could explain this discrepancy. Firstly, the regulation of IL-8 can occur at the post-transcriptional level [27]; thus, even if NF-κB activity is inhibited, the stability and translation efficiency of IL-8 mRNA may be maintained or enhanced by other regulatory proteins. Secondly, alternative signaling pathways, such as MAPK/ERK and JNK, might compensate for the inhibition of NF-κB, sustaining IL-8 production [28]. Thirdly, the IL-8 gene is regulated by multiple transcription factors, including AP-1 and C/EBP, which can drive its expression independently of NF-κB inhibition [29,30]. In addition, recent studies have identified policosanols (PCs) as another class of bioactive compounds present in *C. sativa* extracts. Policosanols, which are long-chain aliphatic alcohols, have been shown to exhibit significant antioxidant and anti-inflammatory activities. These compounds are typically derived from a waxy material obtained through the supercritical fluid extraction of hemp inflorescences. In vitro assays have demonstrated that PCs can reduce intracellular reactive oxygen species production, inhibit NF-κB activation, and decrease the activity of neutrophil elastase, suggesting their potential role in modulating oxidative stress and inflammatory responses. The presence of policosanols in CSE could potentially contribute to the overall anti-inflammatory and antioxidant effects observed in this study [31].

Future studies should focus on elucidating the synergistic and antagonistic interactions between cannabinoids and other phytochemicals in CSE. Investigating the roles of minor cannabinoids, terpenes, and cannflavins and their interactions with major cannabinoids like CBD and THC will provide deeper insights into the full spectrum of anti-inflammatory actions. Additionally, exploring the effects of CSE in vivo and in clinical settings will be crucial for translating these findings into therapeutic applications.

## 4. Materials and Methods

### 4.1. Plant Material and Isolation of Compounds

The *Cannabis sativa* oil extract (CSE) is a commercially standardized extract derived from the aerial parts of *Cannabis sativa* L. and provided by LINNEA SA (Riazzino, Switzerland) (linnea.ch and linneacannabinoids.ch). CSE was achieved through ethanol extraction and extended decarboxylation to convert cannabinoid acids into their neutral counterparts, followed by the replacement of the solvent with medium-chain triglycerides (MCT) (Batch No. 74719004). A purification phase was completed by standardizing the phytocomplexes. CSE was composed of 5% CBD and contained less than 0.2% THC.

The fractionation of the aerial parts of *Cannabis sativa* L. was achieved by the following procedure.

*Cannabis sativa* was extracted with 6 masses of hexane for two hours at boiling temperature. After filtering the solvent, the extraction was repeated twice under the same conditions using fresh hexane. The three extracts were collected, and hexane was removed by vacuum distillation. The dry extract was dissolved in approximately 18 masses of ethanol, and the solution was boiled for 20 h to complete the decarboxylation of the cannabinoids. Ethanol was removed under vacuum to obtain the first extract (see Fraction A in Table 1). Cannabis was dried under a vacuum at 40 °C for 4 h to remove the solvent. The dried cannabis was extracted again with acetone using the previously described procedure. The obtained extract was named Fraction B (Table 1).

The extraction procedure was repeated twice with ethanol and methanol to obtain Fractions C and D (see Table 1).

Additionally, purified forms of different compounds from *Cannabis sativa* aerial parts, Cannflavin A, and canniprene were also provided by LINNEA SA: (−)-cannabidiol, cannabigerol, (−)-cannabichromene, cannabinol, and (−)-trans-Δ^9^-THC (Cod. T-005) was acquired from Merck Life Science S.r.l. (Milan, Italy).

### 4.2. Phytochemical Analysis

Cannabidiol and other cannabinoids are identified and quantified with a validated in-house HPLC method (Linnea SA). The method includes an identification standard prepared internally containing the main cannabinoids (CBDV, CBDVA, THCV, CBG, CBDA, CBGA, CBN, CBC, THCA, Merck Life Science S.r.l., Milan, Italy; THC, Delta-8-THC, LGC Standards GmbH, Wesel, Germany) and a quantification standard containing CBD (Linnea SA). CBD is quantified directly against its own standard, while the other cannabinoids are quantified against CBD using appropriate conversion factors.

The different terpenes were identified and quantified using a validated GC method (Linnea SA), which allows the identification and quantification of more than 40 analytes using an identification standard prepared with the main terpenes of interest and an alpha-pinene quantification standard (Merck Life Science S.r.l. Milan, Italy).

Cannflavin A, Cannflavin B, and canniprene were identified and quantified using an R&D in-house HPLC method (Linnea SA). Canniprene and Cannflavin A were evaluated directly against their own standards (Linnea SA), while Cannflavin B was quantified against Cannflavin A, taking into account the difference in molecular weight.

### 4.3. Cell Culture Procedures

A spontaneously immortalized human keratinocyte line (HaCaT) was acquired from Cell Line Service GmbH (Eppelheim, Germany). These cells were cultured in DMEM (Gibco, Life Technologies, Monza, Italy) supplemented with 10% heat-inactivated fetal bovine serum (Euroclone S.p.A., Milan, Italy), 2 mM L-glutamine (Gibco, Life Technologies, Monza, Italy), and antibiotics (100 U/mL penicillin and 100 mg/mL streptomycin; Gibco, Life Technologies, Monza, Italy) and maintained at 37 °C in a 5% CO_2_ humidified atmosphere. Cells at 80–90% confluence were sub-cultured every four days using 0.25% trypsin-EDTA (Gibco, Life Technologies, Monza, Italy), with the appropriate number (1.5 × 10^6^ cells/flask) transferred to new flasks (Euroclone S.p.A., Milan, Italy) to maintain growth, and 60,000 cells allocated to 24-well plates (DB FalconTM; Corning Life Sciences, Amsterdam, The Netherlands) for assays.

### 4.4. Cell Treatment and Assays

After a 72-h growth period, HaCaT cells were treated with CSE, fractions, or isolated compounds and a pro-inflammatory stimulus (TNFα, 10 ng/mL) dissolved in DMEM (Gibco, Life Technologies, Monza, Italy) supplemented as previously described, without adding serum. Following a 6-h treatment period, the culture medium was collected and stored at −20 °C until analysis or cell lysates were directly subjected to relative measurements. EGCG at a concentration of 20 μM was employed as a benchmark inhibitor of skin inflammation [18].

### 4.5. Cytotoxicity Assay

The structural integrity of the cells pre- and post-treatment was evaluated using light microscopy. For cytotoxicity assessment, we employed the dimethylthiazol-2-yl-2-5-diphenyltetrazolium bromide (MTT) assay (Sigma-Aldrich, Milan, Italy) [32] to evaluate the metabolic activity of mitochondria in treated cells. Post-treatment, the cells were incubated with MTT solution to induce formazan formation (30-40 min), which was then dissolved using a mixture of isopropanol and DMSO (90:10) for spectroscopic analysis at 570 nm (Envision, PerkinElmer, Waltham, MA, USA). Subsequent experiments for CSE, fractions, and pure compounds considered only non-toxic concentration ranges determined by the MTT assay.

### 4.6. Measurement of NF-κB Activity

HaCaT cells were plated in 24-well plates (DB Falcon^TM^; Corning Life Sciences, Amsterdam, The Netherlands) at a concentration of 60,000 cells per well and incubated for 72 h. Subsequently, these cells underwent transient transfection using the lipofectamine method with a reporter plasmid containing the luciferase gene linked to a promoter with three κB responsive elements (NF-κB-LUC, 250 ng/well). The plasmid NF-κB-LUC was kindly provided by Dr. N. Marx is from the Department of Internal Medicine-Cardiology at the University of Ulm, Germany. Following overnight incubation, the cells were treated with CSE, fractions, or specific compounds along with TNFα at a concentration of 10 ng/mL for 6 h.

Luciferase activity within the cells was measured using Britelite^TM^ Plus reagent (PerkinElmer, Waltham, MA, USA) following the guidelines provided by the manufacturer. Luminescence, resulting from the interaction between luciferase and luciferin, was detected using a Victor X3 spectrophotometer (PerkinElmer, Waltham, MA, USA). The data were quantified and reported as mean ± SEM from at least three separate experiments. Epigallocatechin-3-O-gallate (EGCG) at a concentration of 20 μM was employed as a benchmark inhibitor in these assays.

### 4.7. Measurement of IL-8 Release

HaCaT cells were cultured in 24-well plates (DB Falcon^TM^; Corning Life Sciences, Amsterdam, The Netherlands) at a density of 60,000 cells per well and incubated for 72 h. Subsequently, these cells were exposed to CSE, fractions, or isolated compounds along with TNFα (10 ng/mL) as a pro-inflammatory agent for 6 h. Following treatment, the culture medium from each well was harvested and stored at −20 °C until analysis. The release of IL-8 was quantified using an ELISA kit supplied by PeproTech (London, UK). Specifically, corning 96-well EIA/RIA plates from Sigma-Aldrich (Milan, Italy) were prepared by overnight coating at room temperature with the specific antibody provided in the kit. IL-8 levels were determined by the absorbance of the colorimetric reaction involving horseradish peroxidase enzyme and the 3,3′,5,5′-tetramethylbenzidine substrate (Merck Life Science S.r.l., Milan, Italy), with absorbance readings taken at 450 nm for 0.1 s using a Victor X3 spectrophotometer (PerkinElmer, Waltham, MA, USA). The concentration of IL-8 was calculated from a standard curve included in the ELISA kit, ranging from 8 to 1000 pg/mL. Data are presented as mean ± SEM from a minimum of three independent experiments. EGCG (20 μM) was used as a control inhibitor of IL-8 release.

### 4.8. Statistical Analysis

Statistical evaluations were performed on data expressed as mean ± SEM from at least three independent experiments using ANOVA, followed by the Holm-Šídák post-test for multiple comparisons. Statistical significance was set at *p* < 0.05, and the IC_50_ values were computed using GraphPad Prism software version 9.0 (GraphPad Software Inc., San Diego, CA, USA).

## 5. Conclusions

In conclusion, the results of this study on CSE underscore the significant role of CBD in exerting anti-inflammatory effects in human keratinocytes. However, to achieve superior inhibitory outcomes, it is crucial to consider not only the presence of other bioactive molecules but also their specific ratios in the extract. This highlights the importance of utilizing standardized extracts to obtain reproducible data, given the wide variability in the response to these compounds.

## Figures and Tables

**Table 1:** Chemical composition of the CSE fractions. The table lists the percentages of cannabinoids, terpenes, and cannflavins in each fraction (a–d). Fraction a is the most lipophilic, while Fraction d is the most polar.

				Cannabinoids (%)											Canniprene (%)	Terpenes (%)	Cannflavins (%)	
Polarity	Fraction	Weight (g)	Yield (%)	CBD	CBDA	CBDV	CBDVA	CBDC4	THCV	CBG	CBGA	CBC	THC	THCA	Total	Total	Cannflavin A	Cannflavin B
low	Fraction a	19.37	6.46	28.0	0	0.3	0.1	0	0	0.7	0.1	1.0	1.0	0.1	0.4	1.8	0.1	0
↓	Fraction b	6.73	2.40	13.0	0	0.3	0.2	0	0	1.0	0.1	0.6	0	0	0.3	0.2	0.4	0.3
↓	Fraction c	9.69	3.54	0	0	0	0	0	0	0	0	0	0	0	0	0.1	0	0
high	Fraction d	13.72	5.19	0	0	0	0	0	0	0	0	0	0	0	0	0.2	0	0

**Table 2:** Quantification of major cannabinoids and terpenes in the CSE. The table provides the percentage of each identified compound in the whole extract. n.d., not detectable.

	**Cannabinoids** **(%)**											**Canniprene** **(%)**	**Cannflavins** **(%)**	
**Batch Number**	**CBD**	**CBDA**	**CBDV**	**CBDVA**	**CBDC4**	**THCV**	**CBG**	**CBGA**	**CBC**	**THC**	**THCA**	**Total**	**Cannflavin A**	**Cannflavin B**
CM5 74719004	4.836	0.02	0.02	n.d.	0.02	n.d.	0.14	n.d.	0.24	0.22	n.d.	0.0165	0.011	0.004
	**Terpenes** **(%)**													
**Batch Number**	**γ-terpineol**	**β-caryophyllene**	**α-humulene**	**Farnesene isomer 1**	**Farnesene isomer 2**	**Farnesene isomer 3**	**Caryophillene oxide**	**α-bisabolol**	**Total**					
CM5 74719004	0.031	0.050	0.012	0.010	0.007	0.015	0.015	0.011	0.151					

## Data Availability

Data is contained within the article.

## Supplementary Materials

The following supporting information can be downloaded at: https://www.mdpi.com/article/10.3390/ph18050647/s1, Figure S1: Cytotoxicity of pure cannabinoids and terpenes in HaCaT cells (A–G). HaCaT cells were treated with pure cannabinoids or terpene mix (TER) at two different concentrations in the presence of TNFα for 6 h. The viability of cells was measured by MTT assay. The results are presented as the mean ± SEM of three experiments (*n* = 3) and expressed as the relative percentage compared to stimulus (black bar), which was arbitrarily set to 100%.
