# Supplementary material for: Beyond Cannabidiol: The Contribution of Cannabis sativa Phytocomplex to Skin Anti-Inflammatory Activity in Human Skin Keratinocytes
Source: Pharmaceuticals (Basel). 2025 Apr 28;18(5):647. doi: 10.3390/ph18050647 (PMC12114505; doi:10.3390/ph18050647)
Supplement: Supplementary file 1 [file pharmaceuticals-18-00647-s001.zip › pharmaceuticals-3566464-supplementary.pdf]

Supplementary materials

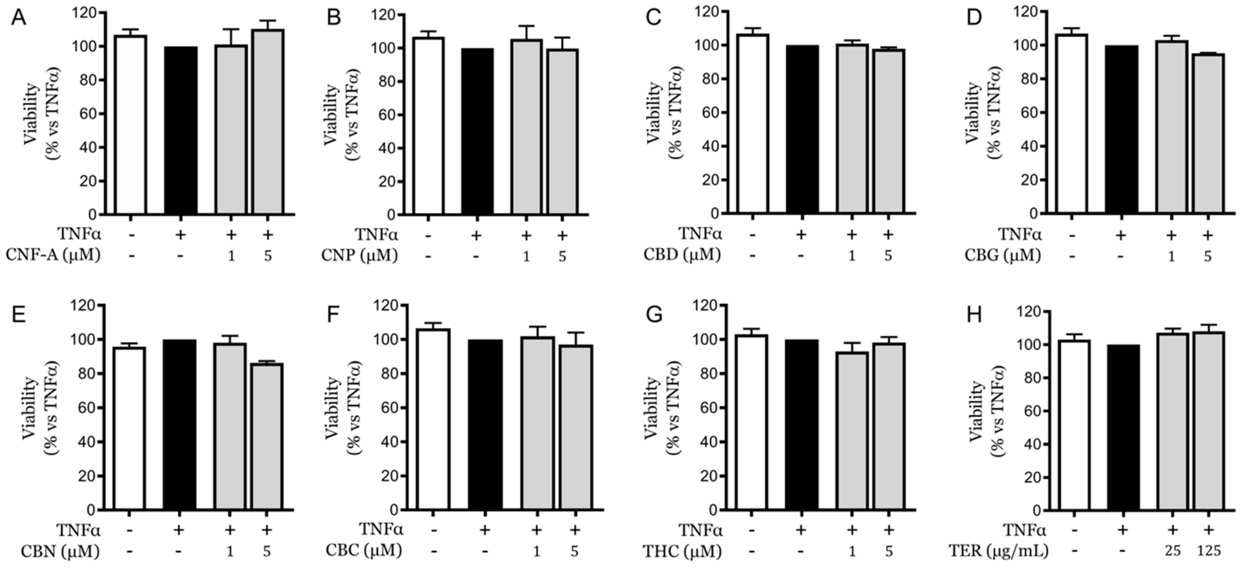

Figure S1. Cytotoxicity of pure cannabinoids and terpenes in HaCaT cells (A–G). HaCaT cells were treated with pure cannabinoids or terpene mix (TER) at two different concentrations in the presence of TNFα for 6 hours. The viability of cells was measured by MTT assay. The results are presented as the mean ± SEM of three experiments (*n* = 3) and expressed as the relative percentage compared to stimulus (black bar), which was arbitrarily set to 100%.
